# Evaluation of Upper Limb Joint Contribution to Racket Head Speed in Elite Tennis Players Using IMU Sensors: Comparison between the Cross-Court and Inside-Out Attacking Forehand Drive

**DOI:** 10.3390/s22031283

**Published:** 2022-02-08

**Authors:** Bruno Pedro, Filipa João, Jerusa P. R. Lara, Silvia Cabral, João Carvalho, António P. Veloso

**Affiliations:** 1 Laboratório de Biomecânica e Morfologia Funcional, CIPER, Faculdade de Motricidade Humana, Universidade de Lisboa, 1499-002 Cruz Quebrada-Dafundo, Portugal; bmpedro@fmh.ulisboa.pt (B.P.); filipajoao@fmh.ulisboa.pt (F.J.); jprlara@yahoo.com.br (J.P.R.L.); scabral@fmh.ulisboa.pt (S.C.); jhcarva@ualg.pt (J.C.); 2 Reference Center in Sports Sciences—SESI, 05303-902 São Paulo, Brazil; 3 Departamento de Ciências Exatas, Naturais e do Desporto, Escola Superior de Educação e Comunicação, Universidade do Algarve, 8005-139 Faro, Portugal

**Keywords:** tennis forehand, 3D joint kinematics, upper limb contribution, racket velocity

## Abstract

This study aimed to quantify and compare the upper limb angular kinematics and its contributions to the racket head speed between the cross-court (CC) and inside-out (IO) attacking tennis forehand of elite tennis players in a competitive environment. A new approach was used to study the forehand drive with mini-inertial sensors of motion capture to record the kinematic data. Six strokes in each direction per participant (72 shots in total) were chosen for analysis. Upper limb kinematics were calculated in the Visual 3D platform (Visual 3D Professional V5.01.21, C-motion, Germantown, MD, USA). The method used to calculate the upper limb’s contributions was performed with MATLAB software and used the segment’s (upper arm, forearm and hand) angular velocities and their respective displacement vectors obtained through the inertial sensors. Upper limb kinematics demonstrated a higher shoulder rotation in the IO direction with significant differences at the end of the backswing, which could be a key factor in distinguishing the two directions of the shot. Results also demonstrated that the horizontal flexion of the upper arm (around the shoulder joint) was primarily responsible for the racket velocity in the anteroposterior direction (48.1% CC and 45.2% IO), followed by the extension of the forearm (around the elbow joint) (17.3% CC and 20.9% IO) and the internal rotation of the upper arm (around the shoulder joint) (15.6% CC and 14.2% IO). No significant differences were shown in the contributions of upper limbs to the racket head velocity between the two directions of the shot. Tennis coaches and players should develop a specific training programme to perform higher angular velocities in these specific joint rotations.

## 1. Introduction

As the second most important stroke in tennis after the serve [1], with which tennis players constantly try to dominate the point [2], the cross-court (CC) and the down-the line (DL) forehand drive [3,4,5] have been the focus of kinematic studies in tennis and table tennis [6]. The latest studies demonstrated several kinematic differences between the CC and DL direction of the shot, such as the racket velocity, hip alignment, and shoulder alignment [3], and also in the knee and elbow flexion, among others [6], presenting valuable information to tennis coaches.

Although the forehand drive is performed mostly from the forehand side of the court, advanced players are able to cover up to 85% of the court with the forehand and it can be used to produce more “winners” shots [7]; thus, it can be used from both sides, and used as a tactical advantage to the shots on the left side of the court [8]. This shot, the so-called inside-out (IO) forehand, can be defined as a forehand played from the backhand side diagonally to the opponent’s backhand [9]. To our knowledge, kinematic studies on the forehand drive have analysed the CC and DL forehand drive, whereas no study has investigated the differences between the CC and the IO forehand.

Researchers agree that understanding the mechanics of the movement is essential to develop racket velocity and also to minimise the risk of injury for the athlete [10]. Thus, the speed of the racket head at impact is critical in tennis and can be varied by the players, particularly via the upper limb segments. Furthermore, the role of the angular velocity vectors of the upper arm, forearm, and hand in generating maximum racket impact speed can produce valuable information for those interested in tennis [11]. Studies have been undertaken on the contributions of the individual segments’ rotations of the upper limb to the racket head velocity in the tennis forehand between players with different grip positions [12], in the tennis serve [11] and in the squash forehand [13]. However, there is no reference to the contributions describing different directions of the shot. Moreover, none of these studies were performed with opposition on the other side of the court. This condition can assume particular importance in the execution of the movement due to the fact that the decision to place the ball cross court or down the line depends on the perception of the opponent’s position on the court.

Later studies and the majority of kinematic studies have been conducted primarily using optical systems [11,12,13,14] and, despite their accuracy [15], these systems require a considerable effort to capture tasks outside a laboratory [16]. Alternatively, inertial measurement unit systems (IMUs) provide a lighter, portable, and easier-to-use system for capturing data outside of a laboratory [17], and an accurate and reliable method to study human movement [18]. In the tennis forehand drive, in particular, IMUs presented very good agreement and reliability for the majority of joint angles [19] when compared with an optical system. Good agreement was also found for upper limbs during simulated swimming [20].

With this in mind, and considering the significant differences shown between the CC and DL in previous studies [3,4,5,6], there is a lack of knowledge about the kinematic variables and the contributions of the upper limb that can distinguish the offensive tennis forehand drive when players play in the CC and the IO directions. Therefore, this study aimed to quantify and compare the kinematics and the contributions of the upper limb (upper arm, forearm, and hand) segments’ rotation to the racket head velocity in a tennis forehand during an attacking situation of elite tennis players, when players performed the forehand drive in the CC and the IO directions, with opposition players creating a more representative situation. We hypothesised that the two directions of the shot (CC and IO) would present (a) different upper limb angular kinematics, and (b) different contributions of the upper limbs to the racket head velocity.

## 2. Materials and Methods

### 2.1. Participants

A convenience sample of six elite male right-handed tennis players (age 21 ± 4.2 years, height 178.2 ± 2.9 cm, mass 73.0 ± 1.8 kg), volunteered to participate in the study. Three had Association of Tennis Professional (ATP) rankings (630, 1156, and 1520, respectively) and the other three had national rankings in Portugal (4, 44, and 69, respectively). Two other tennis players with national rankings collaborated in the study, one male and one female (age 19.5 ± 0.7 years, height 168.5 ± 9.2 cm, mass 65 ± 8 kg) to create an opposition situation. All participants were free from injuries, practised regularly, competed at national and international level, and provided written informed consent to participate in this study, which was approved by the Institution’s Ethics Committee and meets the declaration of Helsinki.

### 2.2. Testing Protocol

**Figure 1:**
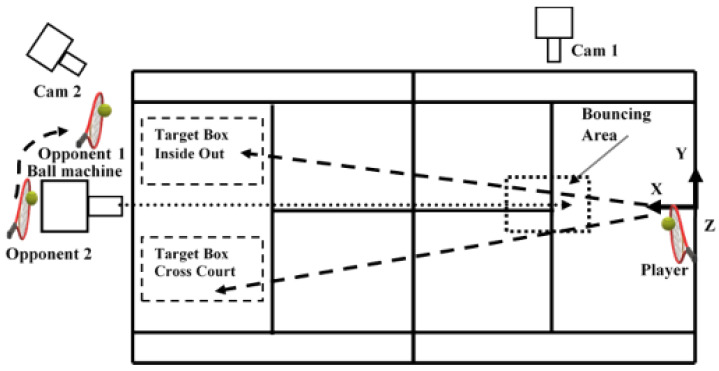
Testing environment. (1) One Lobster ball machine with the projection line toward the bouncing area, (2) one Qualisys Oqus 210c camera (Cam 1), (3) one Panasonic digital video camera (Cam 2), (4) coordinate system, (5) stroke direction, (6) bouncing area, (7) target boxes for the CC and IO direction forehands, (8) participant with Xsens IMUs, (9) opponents 1 and 2.

Before testing, all the participants performed the necessary warm-up and hit as many practice shots as they wanted [4]. A ball machine (Lobster—Phenom Electric Tennis Ball Machine D641, 437, North Hollywood, CA, USA) projected new tennis balls (Dunlop All Court) with a controlled horizontal velocity (24.5 m/s) similar to that of other studies [3,4], to a bouncing area (Figure 1) where the players were able to play an offensive forehand. The opponent assumed different positions on the court and the player had to place the ball on the opposite side. Participants were encouraged to hit the ball as they would in a match when attempting to hit a winner. One Panasonic HC-V10 digital video camera (Figure 1, cam 2) operating at 60 Hz captured the placement of the shot. The shots were considered valid, “in”, when landed inside the CC and IO target boxes (3 × 4.5 m) (Figure 1) [21]. Six valid shots in each direction (CC and IO direction) inside the target area were chosen for analyses (72 forehand shots over all participants). Data were analysed during the acceleration phase, which was determined from the first forward movement of the racket shaft in the anteroposterior direction (X) (Figure 1) until ball impact, defined as the instant where the ball–racket contact occurred.

### 2.3. Instrumentation

The data were captured at 120 Hz with 17 IMU sensors (Xsens MVN Technologies, Enschede, The Netherlands) [22], using the MVN Studio Pro software, and were continuously updated using a biomechanical model of the human body [16]. The accuracy of these IMUs showed an excellent coefficient of multiple correlation values (CMC ≥ 0.95) for the majority of the variables of the upper limb (shoulder, elbow, and wrist) [19]. The placement of sensors in each segment was intended to be over the bones whenever possible to reduce soft tissue artefacts [23]. Calibration procedures were performed to align the sensors with the body alignment and to determine the segment’s length [24]. One Qualisys video camera, model Oquos 210c (Qualisys AB, Gothenburg, Sweden) operating at 240 Hz in Qualisys Track Manager (version 2.10., Qualisys AB, Gothenburg, Sweden), was synchronised with the IMUs to identify the instant of impact.

### 2.4. Data Processing

**Figure 2:**
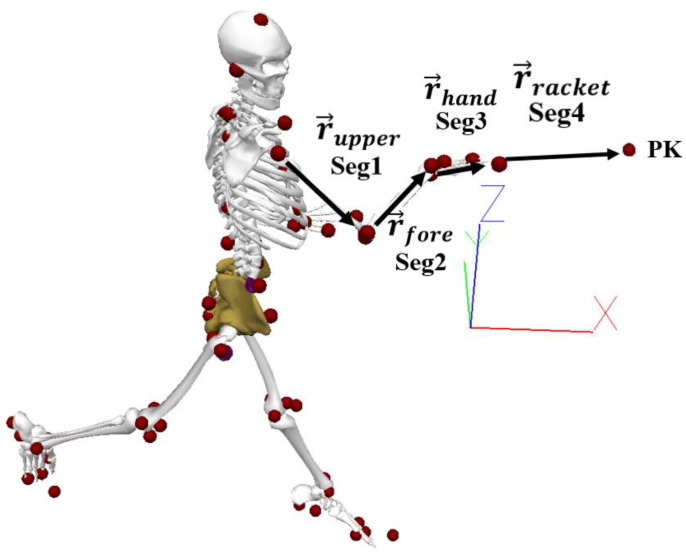
Participant’s virtual markers and derived vectors. Seg1 (segment 1: upper arm), Seg 2 (segment 2: forearm), Seg 3 (segment 3: hand), Seg 4 (segment 4: racket), Pk (centre of the racket).

The three-dimensional coordinates of virtual markers representing bony landmarks in each segment (Figure 2) (created in the MVN Studio^TM^ Pro software based on the IMU model) were exported to C3D format. In Visual 3D software (Visual 3D Professional V5.01.21, C-motion, Germantown, MD, USA), these virtual markers were used for the reconstruction of a biomechanical model with three degrees of freedom (DOF) for the shoulder, two DOF for the elbow, two DOF for the wrist, three DOF for the trunk, and three DOF for the pelvis. The rigid segments were reconstructed as follows: upper arm (projected from the shoulder joint centre to the mid-point between the lateral and medial epicondyle of the humerus); forearm (projected from the mid-point between the lateral and medial epicondyle of the humerus to the mid-point between the lateral point of the radial styloid and the medial point on the ulnar styloid); hand (projected from the mid-point between the lateral point of the radial styloid and the medial point on the ulnar styloid to the mid-point between the base of the second and fifth metacarpal proximal phalanges); trunk (projected from the mid-point between the C7 and the suprasternal notch and the medial point between T8 and PPX); and pelvis (projected from the mid-point between iliac crests and the medial point between right and left great trochanters), with a length and an imported local coordinate system from MVN Studio^TM^ Pro software. Kinematic variables of interest from the dominant upper limb during the forehand drive were as follows: shoulder alignment with the baseline (shoulders parallel with the baseline presented an angle of 0° and when perpendicular with the baseline presented an angle of 90°); separation angle (difference between shoulders and pelvis in the transverse plane); shoulder flexion/extension; shoulder abduction/adduction; shoulder internal/external rotation; elbow flexion/extension; elbow pronation/supination; wrist flexion/extension; and wrist abduction/adduction. Joint angles were calculated using a medio-lateral, anteroposterior, axial Cardan sequence [25]. All joint angles were time normalised to the period between the first movement of the racket shaft in the direction of the shot and the impact.

For the contribution of segments’ rotations to the racket head velocity, the centre of the racket (PK) was determined by measuring the distance from the centre of the wrist to the centre of the racket head (Figure 2). The kinematic variables of the centre of the racket and angular velocity, proximal end position, and distal end position from upper limbs (upper arm, forearm, and hand) were exported from Visual 3D (Visual 3D Professional V5.01.21, C-motion, Germantown, MD, USA) to MATLAB (R2010A). The racket head velocity contribution of each of the three segments of the upper limb was determined by orthogonal unit vectors to define each of the reference frames [26]. The contributions of the upper limb kinematics were described according to the anatomical movements as flexion/extension, abduction/adduction, and internal/external rotations for the upper arm (around the shoulder joint), although we reported the combined contribution of the upper arm flexion and abduction with respect to racket head speed, as in a previous study [11]; pronation/supination and flexion/extension for the forearm (around the elbow joint); and palmar flexion/extension and radial/ulnar flexion for the hand (around the wrist joint). Calculations of the upper limb segments for the absolute angular velocity vectors were used to calculate the relative angular velocity vectors of upper arm segments with respect to the proximal attachment. Then, the angular velocities of each joint were resolved to the scalar (dot) product between the relative angular velocity vectors and their appropriate unit vector. To define the contributions of the segments to the racket head velocity in the direction of the movement, the vector cross product between the anatomical angular velocity vectors and their respective positions vectors to the centre of the racket head was computed (Figure 2) [26]. To determine the percentage contributions of each segment rotation to the racket velocity, the velocity was considered as 100%, and each rotation as a percentage of that 100%.

The velocity in the middle of the racket (*V_k_*) (Figure 2) was calculated with the linear velocities of the upper limbs and expressed as:

(1) $${\vec{V}}_{k}={\vec{V}}_{g}+({\vec{W}}_{1}\times {\vec{r}}_{upper})+\left({\vec{W}}_{2}\times {\vec{r}}_{fore}\right)+({\vec{W}}_{3}\times {\vec{r}}_{hand})$$

where ${\vec{V}}_{g}$ is interpreted as the linear velocity contribution that the legs and torso make to develop the racket head velocity, ${\vec{W}}_{1,2,3}$ are the absolute angular velocities of the three segments, and ${\vec{r}}_{upper,fore,hand}$ vectors define the segments’ length. Knowing the relation between absolute and relative velocity:

(2) $$W_{21}=W_{2}-W_{1}$$

(3) $$W_{31}=W_{3}-W_{2}$$

where *W*_21_ are the relative velocities of the forearm segment relative to the arm and *W*_31_ are the relative velocities of the hand relative to the forearm, and relative velocities are expressed in the global reference system. In the algorithm presented in [27], the rotation in the y-axis (adduction and abduction) of the elbow and the rotation in the z-axis (internal and external) of the hand are equal to zero.

$$W_{1}=\left[W_{1X},W_{1Y},W_{1Z}\right]\;(\mathrm{absolute}\;\mathrm{velocity}\;\mathrm{of}\;\mathrm{the}\;\mathrm{upper}\;\mathrm{arm})$$

$$W_{21}=\left[W_{21X},0,W_{21Z}\right]\;(\mathrm{relative}\;\mathrm{velocity}\;\mathrm{of}\;\mathrm{the}\;\mathrm{forearm})$$

$$W_{31}=\left[W_{31X},W_{31Y},0\right]\;(\mathrm{relative}\;\mathrm{velocity}\;\mathrm{of}\;\mathrm{the}\;\mathrm{hand})$$

The contribution of the separate anatomical rotations  (W) can be determined with the following equation:

(4) $${\vec{V}}_{k}={\vec{V}}_{g}+\{({\vec{W}}_{1X}+{\vec{W}}_{1Y}+{\vec{W}}_{1Z})\times {\vec{r}}_{upper-pk}\}\;+\{({\vec{W}}_{21X}+{\vec{W}}_{21Z})\times {\vec{r}}_{fore-pk}\}+\{({\vec{W}}_{31X}+{\vec{W}}_{31Y})\times {\vec{r}}_{hand-pk}\}$$

where ${\vec{V}}_{g}$ is the linear velocity of legs and torso, ${\vec{W}}_{1X,21X,31X}$ are the angular velocities of flexion and extension, ${\vec{W}}_{1Y,31Y}$ are the angular velocities of adduction and abduction, ${\vec{W}}_{1Z,21Z}$ are the angular velocities of internal and external rotation of the three segments, and ${\vec{r}}_{upper-pk,fore-pk,hand-pk}$ are the vectors with their origin in the joint and extremity in the middle of the racket [26].

### 2.5. Statistical Analyses

The means of the six repetitions in the CC and IO directions (72 forehand shots) for each participant of the selected kinematic (Table 1) and contribution (Table 2) variables of the upper arm, forearm, hand, and racket were calculated. Data were first tested for normality using the Shapiro–Wilk test (*p* < 0.05). Differences between variables having a normal distribution were accessed with a paired samples t-test and those that deviated from a normal distribution were assessed using a Wilcoxon Matched-Pairs Signed Ranks test to identify significant differences. For the paired *t*-test, the effect size (ES) was calculated as the Cohen’s d, where ≥0.2, ≥0.5, and ≥0.8, represent small, medium, and large effect sizes [27], respectively. For the Wilcoxon Matched-Pairs Signed Ranks test, the level of significance was set at *p* ≤ 0.05 and effect size (r) was defined as small for small effect ≥ 0.1, medium effect ≥ 0.3, and large effect ≥ 0.5 [28]. Significance was set at *p* ≤ 0.05 for all tests and all data are reported as mean ± SD. All statistical analyses were performed using SPSS version 20 (SPSS Inc. Company, Chicago, IL, USA). The kinematic differences between the two shots were also assessed using the one dimensional (1D) statistical parametric mapping (SPM). This statistical procedure identifies where significant differences occur during the entire waveform. More specifically, SPM-1D using a two-tailed paired sample *t*-test was used to compare each joint angle curve between the two directions of the shot from each participant. The SPM-1D method uses random field theory to identify field regions that co-vary significantly with the experimental design [29,30,31]. Differences were considered statistically significant for *p*-values < 0.05. All SPM analyses were performed in Python 2.7 using the open source package located at http://www.spm.1d.org/ (accessed on 20 December 2021) [32].

## 3. Results

**Figure 3:**
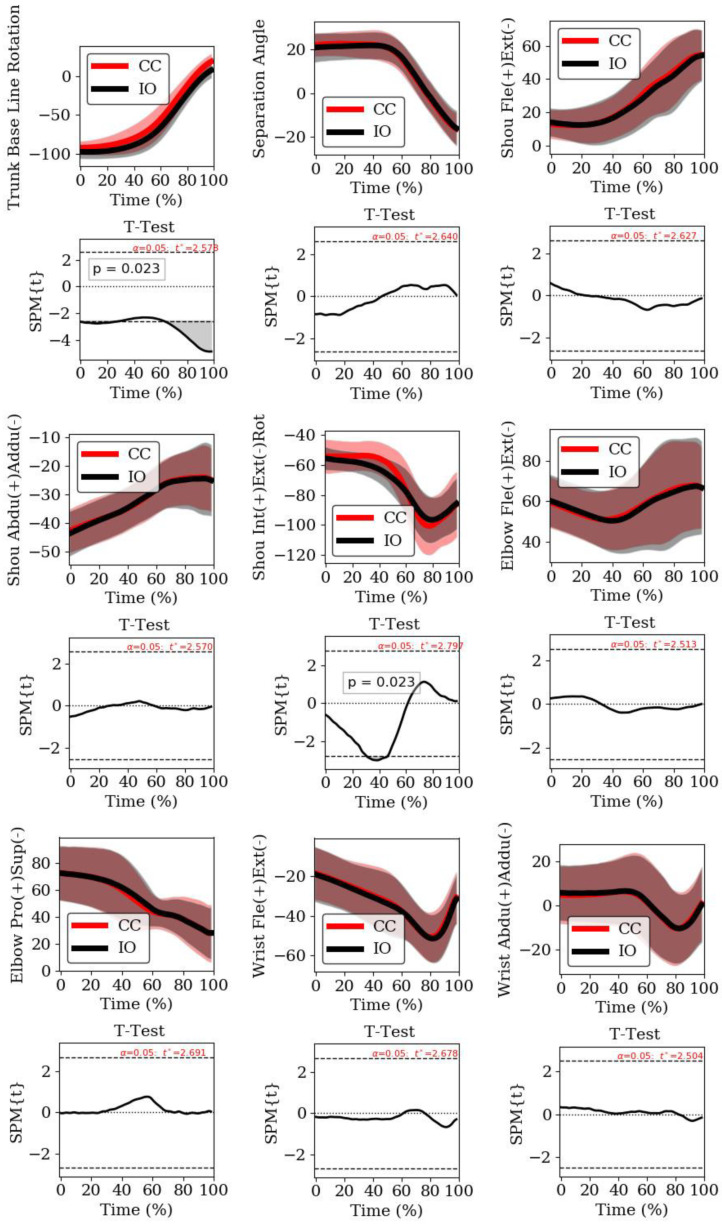
1D-SPM analysis. Joint angles and the respective 1D-SPM results, during the time normalised acceleration phase, between the cross-court (red line) and the inside-out (black line) directions. Trunk baseline rotation, separation angle, shoulder flexion/extension, shoulder abduction/adduction, shoulder pronation/supination, elbow flexion/extension, elbow pronation/supination, wrist flexion/extension, wrist abduction/adduction. The horizontal positive and negative dashed lines indicate the threshold test values (t* values). Grey shaded regions indicate where differences were statistically significant.

Data revealed a normal distribution for all variables except: (a) shoulder alignment with baseline at impact; (b) separation angle at impact; (c) shoulder joint angle flexion/extension at impact; and (d) shoulder joint angle abduction/adduction at impact. Considering the kinematic variables, the only significant differences between the two directions of the shot were in the shoulder alignment to the baseline at the end of backswing, where the shot in the IO direction presented a higher rotation, and at impact, where a higher rotation was found in the CC direction (Table 1). For the remaining joint angles, no significant differences were found between the two directions of the shot (Table 1). The SPM-1D analysis showed significant differences for shoulder alignment with the baseline at the beginning and end of the event, and in shoulder axial rotation at between 32 and 47% of the event (Figure 3), when the SPM trajectory exceeded the threshold (grey area). There were no significant differences and the effect sizes were small in upper limb contributions when comparing both directions (Table 2). In both shots, the order of importance of contributions to the racket head velocity was the flexion/abduction of the upper arm (around the shoulder joint), extension of the forearm (around the elbow joint), internal rotation of the upper arm, shoulder (around the shoulder joint and representing the contribution of the trunk and lower limbs), abduction of the hand, flexion of the hand (around the wrist joint), and the pronation of the forearm (around the elbow joint). The pronation of the forearm had a negative effect on racket head linear velocity, reducing it by up to 2% in the x-direction at impact. At the impact, the linear velocities of the centre of the racket head in the x-direction were not significantly different between the two shots.

## 4. Discussion

The present study aimed to quantify and compare the angular kinematics of the upper limb and its contributions to the racket head velocity during an attacking forehand drive played in the CC and IO directions of elite tennis players using an IMU system. The results showed a higher rotation of shoulders to the baseline at the end of the backswing in the IO direction, which may be a key factor in distinguishing the two directions of the shot. These differences were also corroborated by the SPM analysis, which showed the differences at the end of the backswing and when the ball contact occurred. Considering the contributions of the upper limb rotations to the racket head velocity, the flexion/abduction of the upper arm (around the shoulder joint) was considered the most important contribution to the racket head velocity, followed by the extension of the forearm (around the elbow joint) and the internal rotation of the upper arm (around the shoulder joint). To the best of our knowledge, this is the first kinematic study performed using an opposition player, creating a more representative environment to study these variables.

Limitations of the study are associated with the small number of participants, although this number may be considered reasonable because the level of the participants was elite [33]. Moreover, the motion capture rate may be considered somewhat low (120 Hz); thus, higher capture rate IMUs should be considered for future studies, particularly to ensure that the ball contact is captured.

The calculated velocity of the centre of the racket in this study (Table 2) was somewhat superior to that of tennis players with high-performance forehand drives (≈17.0 m/s) [12], which may reflect the difference between elite and non-elite tennis players. Similar velocities were found at the racket tip with state-ranked tennis players [5], whereas elite tennis players presented a higher mean maximum velocity of approximately 33.1 m/s [4] and approximately 31.1 m/s at impact [3].

At the end of the backswing, the shoulder alignment was found to be almost perpendicular to the baseline (CC: −92.8.8 ± −8.8°, IO: −97.2 ± 8.8°); however, at impact, it was nearly parallel to the baseline (CC: 19.3 ± 9.4°, IO: 8.5 ± 9.4°), which is in accordance with other studies [3,12]. Despite the similarities in the shoulders’ alignment with other studies, significant differences were present between the two directions of the shot (Table 1). Moreover, these significant differences were present during the first phase of the event between 0 and 30% of the forward swing (Figure 3). We consider that these differences at the end of backswing may be associated with the necessary adjustments to a more comfortable alignment to the direction of the shot, and may present an important key factor if noted by players to anticipate the opponent’s shot direction. Regarding the separation angle (CC: 22.1 ± 4.6°, IO: 20.9 ± 6.3°), which is considered an important key factor in stretching appropriate muscles [10] and taking advantage of the stretch-shortening cycle of muscles [2] to support the acceleration phase, it was slightly inferior compared to a fast forehand drive [14], with high-performance tennis players [12] showing approximately 30° between the shoulders and the pelvis. Moreover, higher separation angles were shown in a badminton smash [34]. Nonetheless, our study produced similar results to those from elite tennis players [3], with values between approximately 20 and 25°. The similar separation angles of both directions (Table 1 and Figure 3) indicate that the necessary adjustments are performed by both feet, which maintain a similar separation angle for the different directions of the shot, contrary to the shoulder alignment with the baseline. Regarding the shoulder contribution to the racket head velocity, which represents the contribution of the lower limbs and trunk (CC: 10.4%, IO: 10.6%), there were no significant differences between the two directions of the shot (Table 2), and a similar contribution of approximately 10% was shown with a flat forehand for players with Eastern and Western grips [12], and in the power serve in tennis [11].

The shoulder joint angle flexion/extension was found to be almost aligned with the trunk at the end of the backswing (CC:12.9 ± 8.3°, IO: 14.1 ± 8.2°), similar to high-performance tennis players holding the racket with a western grip [12], moreover, the flexion angle at impact (CC: 54.7 ± 14.1°, IO: 54.3 ± 14.6°) is also similar to that of the latter study. The shoulder abduction maintained a similar value between the end of the backswing and the impact (Table 1). The contribution of the flexion/abduction of the upper arm (CC: 48.1%, IO: 45.2%) was considerably superior (Table 2) when compared with players using an Eastern grip (34.1%) and even more compared with players using a Western grip (20.8%) [12]. One considerable difference between the participants is the fact that the majority used a semi-Western grip, although we do not think this could be related to such a difference. The internal/external rotation of the upper arm presented similar values (CC: −84.9 ± 18.8°, IO: −85.5 ± 19.9°) as those of highly skilled male tennis players [14] for peak external rotation and impact joint angles. By comparison, the 1D_SPM analysis showed significant differences between 33 and 47% of the event (Figure 3), showing an inferior external rotation of the upper arm in the CC direction. The contribution of the internal/external rotation presented inferior importance (CC: 15.6%, IO: 14.2%) compared with a previous study [12], where the authors showed a contribution of approximately 40%. One consideration for this difference may be related to the ball impact height due to the specificity of the stroke inside the tennis court, where a more horizontal movement may be present in our study.

The forearm flexion joint angle showed similar values (Table 1) as those of highly skilled tennis players [14] at the end of the backswing (CC: 59.7 ± 13.3°, IO: 60.4 ± 13.5°) and at the impact (CC: 66.3 ± 18.9°, IO: 66.4 ± 22.9°), whereas the contribution of the extension of the forearm segment (CC: 17.3%, IO: 20.9%) (Table 2) was higher compared with tennis players with Eastern or Western grips, with a contribution between 2.4 and 0.6% for a flat forehand [12]. The axial rotation of the forearm showed a pattern of supination during the acceleration phase, thus showing a negative contribution to the racket head velocity (CC: −2.0%, IO: −0.9%). This is in line with the results of a previous study [12].

The hand segment showed slightly inferior extension values at the end of the backswing (CC: −18.4 ± 13.6°, IO: −19.0 ± 14.2°) and at impact (CC: −30.4 ± 13.3°, IO: −31.3 ± 12.7°) (Table 1) in relation to other studies [12,14], although the contribution of this rotation (CC: 4.5%, IO: 5.3%) is in accordance with a study using high-performance forehand players [12]. For the abduction, the contribution in the present study (CC: 6.1%, IO: 4.9%) (Table 2) was slightly higher compared with participants with an Eastern grip, and inferior to those with a Western grip, during a flat forehand [12].

Although some of the present results were in line with those from the only previous study [12] using this method to calculate the contributions to the racket head velocity in the forehand drive, there were also some discrepancies. Some differences between the two studies should be considered, such as the impact location. In our study, impact occurred inside the court, with players hitting the ball as soon as possible, and in some cases the impact occurred in a position above the net. This may represent a more horizontal movement in comparison with the same stroke performed near the baseline, which may, therefore, present some kinematic differences.

The present findings on the shoulders’ alignment to the baseline may be useful to players and coaches for predicting the direction of the opponent’s forehand, as also seen in table tennis [6], and to identify strategies to disguise their own shot. Moreover, this study showed that these differences can occur within the initial 30% period of the forward swing, thus providing additional important information for tennis coaches and players. Considering the similarities of the contributions to the racket head velocity in both directions of the shot we recommend to coaches and athletes specific strength training to develop high angular velocities in the most important racket head velocity contributions. We also highlight the importance of a physiotherapy program to prevent injury [10] to the external rotators of the shoulder and back muscles, and to compensate for the multiple repetitions of flexion/abduction in the forehand stroke.

## 5. Conclusions

The consistency in joint angles when players hit the ball in the CC and IO directions suggests that highly skilled players have greater motor control consistency, and show differences only in their shoulder alignment with the baseline. This may represent important information for players and coaches, either to anticipate the opponent’s shot or to disguise their own shot. The horizontal flexion of the upper arm (around the shoulder joint) and the extension of the forearm (around the elbow joint) are the most important contributors to the racket head velocity in the forehand drive during an attack situation. Thus, tennis coaches and players should develop a specific training program to develop higher angular velocities in these specific joint rotations. The absence of significant differences in the segment contributions in the two directions of the shot (CC and IO), in terms of most of the joint angles of the upper arm and trunk, demonstrate that an adjustment is made to the feet and the whole body according to the tactical option. Future studies should try to understand the differences in the contributions for different impact locations and levels of expertise.

## Figures and Tables

**Table 1:** Upper limb kinematic variables in cross-court and inside-out directions.

Variables	Cross-Court	Inside-Out	Stat. Test	*p*-Value	ES
Shoulder Alignment Base line					
End of backswing (°)	−92.2 ± 8.8	−97.2 ± 8.8 *	5.966	0.000	0.99
Impact (°)	19.3 ± 9.4	8.5 ± 9.4 *	−5.153	0.000	−2.10
Separation Angle toward dominant arm					
End of backswing (°)	22.1 ± 4.6	20.9 ± 6.3	1.224	0.229	0.20
Impact (°)	−16.5 ± 5.1	−16.5 ± 7.1	−0.094	0.925	−0.04
Shoulder Flexion (+)/extension (−)					
End of backswing (°)	12.9 ± 8.3	14.1 ± 8.2	−1.432	0.161	−0.24
Impact (°)	54.7 ± 14.1	54.3 ± 14.6	0.260	0.796	0.04
Shoulder Abduction (−)/adduction (+)					
End of backswing (°)	−45.1 ± 9.1	−46.6 ± 9.0	1.572	0.125	0.26
Impact (°)	−47.9 ± 4.6	−47.3 ± 7.1	−0.534	0.593	−0.22
Shoulder int. (+)/ext. rotation (−)					
End of backswing (°)	−54.5 ± 9.5	−55.3 ± 8.5	1.112	0.274	0.19
Impact (°)	−84.9 ± 18.8	−85.5 ± 19.9	0.594	0.556	0.10
Elbow Flexion (+)/Extension (−)					
End of backswing (°)	59.7 ± 13.3	60.4 ± 13.5	−1.062	0.295	−0.18
Impact (°)	66.3 ± 18.9	66.4 ± 22.9	−0.074	0.942	−0.01
Elbow Pronation (+)/Supination (−)					
End of backswing (°)	72.8 ± 21.4	72.8 ± 21.2	0.027	0.979	0.00
Impact (°)	28.3 ± 23.0	28.3 ± 20.4	−0.011	0.991	0.00
Wrist Flexion (+)/Extension (−)					
End of backswing (°)	−18.4 ± 13.6	−19.0 ± 14.2	0.496	0.623	0.08
Impact (°)	−30.4 ± 13.3	−31.3 ± 12.7	0.724	0.474	0.12
Wrist adduction (−)/abduction (+)					
End of backswing (°)	4.8 ± 13.7	5.8 ± 13.1	−1.226	0.228	−0.20

Mean and standard deviation for joint angles of upper and trunk from the end of the backswing to impact in the two directions of the shot. Angles are reported in degrees. Zero degrees correspond to the anatomical position. * Significant differences between CC and IO.

**Table 2:** Contribution of the upper limb rotations to the racket head velocity in cross-court and inside-out directions.

Segments	Cross Court		Inside Out				
	M (±SD) (m/s)	Contribut. (%)	M (±SD) (m/s)	Contribut. (%)	*t*-Test	*p*-Value	ES
Shoulder	2.3 ± 0.3	10.4	2.3 ± 0.5	10.6	0.158	0.881	0.06
Upper Arm							
Flex/Ext/Add/Abdu	10.4 ± 4.6	48.1	10.0 ± 3.4	45.2	0.522	0.624	0.21
Intl/Ext Rotation	3.3 ± 2.2	15.6	3.3 ± 2.4	14.2	0.000	1.000	0.00
Forearm							
Flex/Ext	3.7 ± 2.2	17.3	4.6 ± 3.3	20.9	−1.421	0.215	−0.58
Pron/Supi	−0.2 ± 1.4	−2.0	0.1 ± 1.1	−0.9	−0.488	0.646	−0.20
Hand							
Flex/Ext	1.0 ± 0.9	4.5	1.5 ± 1.7	5.3	−0.162	0.878	−0.07
Add/Abd	1.3 ± 1.5	6.1	1.1 ± 1.4	4.9	0.539	0.613	0.22
Centre of racket	21.8 ± 2.2		22.6 ± 1.8		−0.778	0.472	−0.32

Contributions of the shoulder (representing the contribution of the legs and trunk) and upper limb to the linear velocity of the racket in the cross-court and inside-out directions of the shot.

## Data Availability

The data presented in this study are available on request from the corresponding author.
